# Construction and validation of a brain magnetic resonance imaging template for normal older Koreans

**DOI:** 10.1186/s12883-024-03735-8

**Published:** 2024-06-28

**Authors:** Wheesung Lee, Subin Lee, Yeseung Park, Grace Eun Kim, Jong Bin Bae, Ji Won Han, Ki Woong Kim

**Affiliations:** 1 Department of Brain & Cognitive Sciences, Seoul National University College of Natural Sciences, Seoul, Republic of Korea; 2 Department of Neuropsychiatry, Seoul National University Bundang Hospital, Seongnam, Republic of Korea; 3 Department of Psychiatry, College of Medicine, Seoul National University, Seoul, Republic of Korea

**Keywords:** MRI, Brain, Template, Korean, Old

## Abstract

**Background:**

Spatial normalization to a standardized brain template is a crucial step in magnetic resonance imaging (MRI) studies. Brain templates made from sufficient sample size have low brain variability, improving the accuracy of spatial normalization. Using population-specific template improves accuracy of spatial normalization because brain morphology varies according to ethnicity and age.

**Methods:**

We constructed a brain template of normal Korean elderly (KNE200) using MRI scans 100 male and 100 female aged over 60 years old with normal cognition. We compared the deformation after spatial normalization of the KNE200 template to that of the KNE96, constructed from 96 cognitively normal elderly Koreans and to that of the brain template (OCF), constructed from 434 non-demented older Caucasians to examine the effect of sample size and ethnicity on the accuracy of brain template, respectively. We spatially normalized the MRI scans of elderly Koreans and quantified the amount of deformations associated with spatial normalization using the magnitude of displacement and volumetric changes of voxels.

**Results:**

The KNE200 yielded significantly less displacement and volumetric change in the parahippocampal gyrus, medial and posterior orbital gyrus, fusiform gyrus, gyrus rectus, cerebellum and vermis than the KNE96. The KNE200 also yielded much less displacement in the cerebellum, vermis, hippocampus, parahippocampal gyrus and thalamus and much less volumetric change in the cerebellum, vermis, hippocampus and parahippocampal gyrus than the OCF.

**Conclusion:**

KNE200 had the better accuracy than the KNE96 due to the larger sample size and was far accurate than the template constructed from elderly Caucasians in elderly Koreans.

**Supplementary Information:**

The online version contains supplementary material available at 10.1186/s12883-024-03735-8.

## Introduction

Spatial normalization to a standardized brain template is a crucial step in comparing brain magnetic resonance images between individuals or groups. Spatial normalization transforms the original image into a standard template so that the corresponding structures coincide spatially coincided [1]. This process allows magnetic resonance images to maintain individual traits of the brain while reducing variability caused by its shape, volume, or relative orientation [2–4]. However, this process inevitably produces deformation of the original images, and the accuracy of the consequent image processing procedures, such as segmentation of tissues, can be enhanced by reducing the deformation associated with spatial normalization [5, 6].

Deformations associated with spatial normalization can be reduced by using population-matched templates because brain morphology differs considerably between populations [7]. The shape and/or size of the brain are quite different between races [8, 9]. The brains of young Caucasians are longer but narrower than those of young Asians [9]. The shape of the brain is different even between Asians [10]. For example, the brains of Chinese individuals were longer but narrower than those of Indians [10]. Brain morphology is also different between sexes, and it changes with advancing age. The brains of women were smaller but showed a higher percentage of gray matter than men [11]. The volume of gray and white matter decreases with advancing age [12]. The Chinese2020 template developed from Chinese subjects aged 18 to 76 years was shorter in length, width, and height than the Chinese56, which was developed from Chinese subjects aged 21 to 30 years [9, 13]. Therefore, it is crucial to develop population-specific brain templates using sex- and age-balanced datasets.

However, in constructing various Caucasian and Asian brain templates, the sex and/or age of the participants was not properly balanced [10, 13–15]. The MNI152 template, which is the most popular adult brain magnetic resonance image template, was constructed by averaging the magnetic resonance images of 152 normal and younger Caucasian adults (86 men and 66 women aged 18 to 44 years) [3, 14, 16, 17]. Furthermore, psychiatric, neurological, or medical conditions that may influence the brain were not evaluated and/or excluded in the development of most previous brain templates. For example, cognitively impaired but not demented subjects were not excluded in the age-specific templates reported by Fillmore and colleagues [15]. In most studies, neurological or psychiatric disorders were not directly evaluated and excluded based on their lifetime history [9, 10, 13–15].

In this context, we constructed a brain magnetic resonance imaging (MRI) template for older Korean adults (KNE96) [18]. In the KNE96, only healthy and cognitively normal Koreans aged 60 years or older were included through extensive and structured psychiatric, neurological, and physical examinations and laboratory tests. Additionally, the age and sex of the participants were strictly balanced. In each age stratum, 12 men and 12 women were included. The displacement and volumetric change of the brain MRI scans of old Korean adults that were associated with spatial normalization to the KNE96 were significantly lower than those associated with spatial normalization to the MNI152 [18]. However, the KNE96 was constructed using data from older adults and the MNI152 was constructed with data from younger adults. Because age-associated brain atrophy was comparable between East Asians and Caucasians, the difference in the accuracy of spatial normalization between the KNE96 and the MNI152 in our previous study may be attributable to the age differences as well as to the ethnic differences between the templates [19]. Furthermore, the quality of brain MRI templates is considerably influenced by sample size. The small sample size led to high brain template variability. The mean deformation value and the logarithmically transformed Jacobian determinant asymptotically decreased as the sample size increased until the sample size reached 200 [7].

Therefore, in the present study, we constructed a new brain MRI template for older Korean adults (the KNE200) from 200 healthy and cognitively normal Koreans aged 60 years or older (five 5-year age strata, with 20 men and 20 women in each age stratum). We compared its accuracy of spatial normalization with that of the KNE96 and the template for older Caucasians constructed by Fillmore and colleagues (OCF) [15].

## Methods

### Study participants

We constructed two datasets: a development dataset consisting of brain magnetic resonance images of 200 right-handed, healthy, cognitively normal Koreans aged 60 years or older, and a validation dataset consisting of 100 right-handed, healthy, cognitively normal Koreans aged 60 years or older. The participants included in both datasets were selected from two population-based prospective cohort studies (the Korean Longitudinal Study on Healthy Aging [KLoSHA] and the Korean Longitudinal Study on Cognitive Aging and Dementia [KLOSCAD]) and the visitors to the dementia clinic of Seoul National University Bundang Hospital [20, 21]. In 2005, the Korean Longitudinal Study on Health and Aging (KLoSHA) was initiated as a population-based prospective cohort study, with the aim of investigating health, aging, and common geriatric diseases among Korean elders aged 65 and over. This study randomly selected 714 community-dwelling Koreans from Seongnam, South Korea, and has been tracking them from 2005 to 2020. The baseline study was conducted from September 2005 to September 2006 in Seongnam, with subsequent follow-up studies planned at 4-year intervals indefinitely. KLoSHA was designed to estimate the prevalences and risk factors of common geriatric disorders among Korean elders, as well as to establish normative data for neuropsychological measures, general health parameters, and laboratory tests. Beyond offering comprehensive epidemiological data on the health status and common geriatric disorders of Korean elders, KLoSHA aims to foster comprehensive multidisciplinary and interdisciplinary research on aging and geriatric disorders. Additionally, it seeks to contribute to the formulation of policies and the planning of health management programs and social services in Korea, thereby addressing a critical need in the rapidly aging society [20]. Initiated in 2010, the Korean Longitudinal Study on Cognitive Aging and Dementia (KLOSCAD) represents the inaugural nationwide, multi-center, population-based prospective cohort study in South Korea, targeting the geriatric population. Initially, a random sample of 6,818 residents aged 60 years or older from 13 districts across South Korea was selected in October 2010. Subsequent follow-up evaluations have been systematically undertaken biennially. The study’s comprehensive assessments encompass the diagnosis of cognitive disorders, a detailed neuropsychological battery, assessments of behavioral and psychological symptoms of dementia, evaluations of activities of daily living, physical and neurological examinations, laboratory tests, lifestyle assessments, quality of life measurements, and identification of death events. The KLOSCAD is positioned to contribute robust scientific evidence towards enhancing global and domestic efforts in combating dementia [21]. The details of both studies have been described in our previous studies [20, 21].

The development dataset included 100 men (72.49 ± 8.04 years old) and 100 age-matched women (72.36 ± 7.46 years old). The development dataset consisted of five age strata (60–64 years, 65–69 years, 70–74 years, 75–79 years, and 80 years or above), and each age stratum included 20 men and 20 age-matched women. All participants included in the development dataset were community-dwelling, functioning independently, and cognitively normal, with a Clinical Dementia Rating (CDR) of 0 and a Mini-Mental State Examination (MMSE) score of 24. They had no major psychiatric, neurological, or medical disorders that could affect their cognition. All comorbid illnesses were mild and rated as a value of two or lower on the Cumulative Illness Rating Scale (CIRS).

The validation dataset included 50 men (73.39 ± 7.33 years old) and 50 age-matched women (73.10 ± 7.42 years old). The validation dataset consisted of five age strata (60–64 years, 65–69 years, 70–74 years, 75–79 years, and 80 years or above), and each age stratum included 10 men and 10 age-matched women. There were no overlapping participants between the validation dataset and the development dataset. All participants included in the validation dataset were community-dwelling, functioning independently, and cognitively normal, with a CDR of 0 and MMSE score of 24. They also had no major psychiatric, neurological, or medical disorders that could affect cognition.

Geriatric psychiatrists assessed all participants through face-to-face standardized diagnostic interviews using the Korean version of the Consortium to Establish a Registry for Alzheimer’s Disease Assessment Packet (CERAD-K) Clinical Assessment Battery and the Korean version of the Mini International Neuropsychiatric Interview (MINI), and conducted neurological and physical examinations and laboratory tests [22, 23]. We obtained previous histories of mental, neurological, and medical disorders from the participants and/or their family members and quantified the burden of comorbid chronic medical illnesses using the CIRS [22]. Neuropsychologists or trained research nurses evaluated cognitive function using the CERAD-K Neuropsychological Assessment Battery, Digit Span Test, and Frontal Assessment Battery, and handedness using the Edinburgh Handedness Inventory [22, 24, 25]. Thereafter, a panel of geriatric psychiatrists determined the participants’ diagnosis of major psychiatric disorders, including dementia, according to the Diagnostic and Statistical Manual of Mental Disorders, Fourth Edition (DSM-IV) diagnostic criteria, and the global severity of cognitive impairments using the CDR [26].

The study protocol was approved by the Institutional Review Board of Seoul National University Bundang Hospital (IRB number; B-0508/023 − 003 for the KLOSHA and B-0912-089-010 for the KLOSCAD). All participants were fully informed of the study protocol and provided written informed consent by themselves or their legal guardians.

### Image acquisition and processing

We obtained high resolution three-dimensional (3D) T1-weighted spoiled gradient echo (SPGR) magnetic resonance images of all participants using Philips 3.0 Tesla Achieva scanners (Philips Medical Systems; Eindhoven, the Netherlands) with the imaging parameters as follows: acquisition voxel size = 1.0 × 0.5 × 0.5 mm; 1.0 mm sagittal slice thickness with no inter-slice gap; echo time = 4.6 ms; repetition time = 8.1 ms; number of excitations = 1; flip angle = 8°; field of view = 240 × 240 mm; and acquisition matrix size = 175 × 256 × 256 mm in the x-, y-, and z-dimensions.

All data were acquired in Digital Imaging and Communications in Medicine (DICOM) format and then converted into ANALYZE format for analysis using the import function of the Statistical Parametric Mapping software (version 12, SPM12; Wellcome Trust Centre for Neuroimaging, London; https://www.fil.ion.ucl.ac.uk/spm) in MATLAB R2019b (MathWorks Inc., Natick, MA, USA). We reoriented the magnetic resonance images to match the anterior commissural-posterior commissural (AC-PC) line of the brain to the y-axis, the vertical anterior commissural (VAC) line to the z-axis, and the midsagittal plane to the y-z plane. Then we resliced them into an isotropic voxel size of 1.0 × 1.0 × 1.0 mm and removed the inhomogeneous signal intensity using bias field correction. The height, length, and width of the whole brain were determined as the distances between the most inferior to the most superior points, right to left points, and anterior to posterior points using an in-house code run on MATLAB. In addition, the width-to-length (W/L), height-to-length (H/L), and height-to-width (H/W) ratios were calculated.

### Construction of the KNE200 template

We constructed the KNE200 template from 200 high-resolution 3D structural magnetic resonance images of the development dataset using the DARTEL toolbox in SPM12 [27]. We segmented each scan using the ‘Old-segment’ tool in SPM12 and generated rigidly aligned images of each tissue class using the spatial transformation files created from the process. Using the ‘Initial Import’ tool, we aligned the T1 images of all participants together with the three tissue classes so that all scans exist in the same space and have the same voxel size. Each tissue type and T1 image separately underwent a non-linear registration, and the initial template was generated from the mean of the 200 images from the construction group. Deformations from the initial template to the individual images were computed, and the inverse and mean values of these deformations were used to update the template. Although the templates of T1 scans and each tissue types were separately generated, they were aligned to the same space. We repeated this process 36 times with six outer iterations which included six inner iterations. As a result, we obtained six templates from each outer iteration, and the sixth template, the most detailed and crisp template, was chosen as the final template.

### Validation of the KNE200 template

We measured the displacement and volumetric change of every voxel between the original and registered images and compared them between the brain templates [18]. To examine the effect of sample size on the accuracy of spatial normalization, we compared them between the KNE200 and KNE96 templates. To examine the effect of ethnicity on the accuracy of spatial normalization, we compared the age-specific templates of the KNE200 and the OCF templates. The age-specific OCF templates were created consisted of six age strata (60–64 years, 65–69 years, 70–74 years, 75–79 years and 80–84 years, 85–89 years) [15]. Therefore, we averaged the age-specific templates of the 80–84 and 85–89-year-olds to create age-specific templates for those aged 80 years or above for comparison with the KNE200. We registered the magnetic resonance images of the validation dataset to the KNE200, KNE96, and five age-specific templates of the KNE200 and OCF. We then analyzed the deformation of each voxel in the registration process by measuring the change in the location and volume of each voxel between the original and registered images. We measured the magnitude of displacement by calculating the Euclidean distance between the voxel in the original and registered images, and the volumetric change of each voxel by calculating the log of the Jacobian determinant of each voxel between the original and registered images [18, 28]. We constructed maps of the magnitude of the displacement and volumetric changes of each template. Because these maps were created in each template space, we matched the coordinates of these maps by spatially normalizing them to the MNI space using the DARTEL toolbox [27]. To explore the region-specific differences, we segmented these maps using the AAL3 atlas and analyzed the deformation of each anatomical region. Voxels were classified into 166 regions using the AAL3 atlas [29]. Because we excluded small regions that had volumes smaller than 500 mm3, 133 regions were compared. For each region, mean deformation values were calculated. For the log of Jacobian determinants, absolute values were used to compare the means. The log Jacobian values are − 1<, 0, and < 1 if there is volume expansion, preservation, or contraction, respectively [18]. By taking the absolute value, we prevented the values from cancelling each other and measured the mean volumetric change in both directions. To analyze the effect of sample size on the accuracy of spatial normalization, we compared the maps of the magnitude of displacement and the log values of the Jacobian determinants of the KNE200 and KNE96. To analyze the effect of sample ethnicity on spatial normalization, we compared the maps of the magnitude of displacement and the log values of the Jacobian determinants of the five age groups. For both comparisons, we performed a paired t-test with Bonferroni correction, where *p* < 3.13*10 − 4 was considered statistically significant.

## Results

Demographic and clinical characteristics of the development dataset are summarized in Table 1. The development dataset included 100 men and 100 age-matched women, divided into five age groups, each consisting of 20 men and 20 age-matched women. The brains of men were higher, wider, and longer than those of women. However, the width/length, height/length, and height/width ratios were comparable between men and women. Brain sizes and their ratios were also comparable across all five age groups.

**Table 1:** Characteristics of the development dataset

	Age group								Sex		
	60-64^a^	65-69^b^	70-74^c^	75-79^d^	80 + ^e^	*p* ^*^	post-hoc^*^		Men	Women	*p* ^†^
Age (years)^‡^	62.2 ± 1.2	67.5 ± 1.5	72.0 ± 1.3	77.2 ± 1.3	83.5 ± 3.7	< 0.001	a < b < c < d < e		72.5 ± 8.0	72.4 ± 7.5	0.615
Education (years)^‡^	12.0 ± 5.0	13.5 ± 4.2	11.6 ± 5.4	11.2 ± 4.8	9.8 ± 5.1	0.025	e < b		13.0 ± 5.1	10.3 ± 4.6	0.213
MMSE (points)^‡^	27.1 ± 2.4	28.1 ± 1.5	27.6 ± 2.6	26.9 ± 2.2	26.9 ± 2.4	0.08			27.9 ± 1.8	26.7 ± 2.6	0.004
Height (mm)^‡^	98.6 ± 8.3	101.3 ± 8.8	100.5 ± 7.3	101.6 ± 7.6	101.1 ± 10.5	0.519			102.1 ± 8.2	99.1 ± 8.8	0.006
Length (mm)^‡^	160.9 ± 9.4	161.3 ± 8.2	162.6 ± 7.6	163.0 ± 7.2	160.7 ± 10.2	0.689			165.0 ± 7.8	158.4 ± 8.2	< 0.001
Width (mm)^‡^	129.9 ± 8.9	132.5 ± 8.3	132.4 ± 6.5	133.4 ± 5.8	132.9 ± 6.9	0.278			134.9 ± 6.1	129.5 ± 7.3	< 0.001
Width/Length^‡^	0.81 ± 0.01	0.82 ± 0.01	0.82 ± 0.01	0.82 ± 0.01	0.83 ± 0.01	0.419			0.82 ± 0.05	0.82 ± 0.05	0.838
Height/Length^‡^	0.61 ± 0.01	0.63 ± 0.01	0.62 ± 0.01	0.62 ± 0.01	0.63 ± 0.01	0.441			0.62 ± 0.04	0.63 ± 0.04	0.240
Height/Width^‡^	0.76 ± 0.01	0.77 ± 0.01	0.76 ± 0.01	0.76 ± 0.01	0.76 ± 0.01	0.984			0.76 ± 0.05	0.77 ± 0.05	0.191

MMSE, mini mental status examination

Each age group consisted of 20 men and 20 women. All values are presented as mean ± standard deviation

^*^ One-way analysis of variance with Bonferroni post-hoc comparisons

^†^ Paired *t*-test

Demographic and clinical characteristics of the validation dataset are summarized in Table 2. The validation dataset included 50 men and 50 age-matched women, divided into five age groups, each consisting of 10 men and 10 age-matched women. While the height of the brains of men and women were comparable, the brains of men were wider and longer than those of women.

**Table 2:** Characteristics of the validation dataset

	Age group								Sex		
	60-64^a^	65-69^b^	70-74^c^	75-79^d^	80 + ^e^	*p* ^*^	post-hoc^*^		Men	Women	*p* ^†^
Age (years)^‡^	62.5 ± 0.3	69.1 ± 0.1	73.2 ± 0.2	78.3 ± 0.2	83.2 ± 0.5	< 0.001	a < b < c < d < e		73.4 ± 7.3	73.1 ± 7.4	0.847
Education (years)^‡^	14.5 ± 0.8	12.5 ± 1.1	9.0 ± 1.5	11.8 ± 0.9	10.8 ± 1.1	0.011	c < a		13.6 ± 4.8	9.8 ± 4.7	< 0.001
MMSE (points)^‡^	28.2 ± 0.3	28.1 ± 0.4	26.3 ± 0.6	26.5 ± 0.6	25.4 ± 0.8	0.003	e < a,b		27.4 ± 2.5	26.3 ± 2.9	0.054
Height (mm)^‡^	105.5 ± 1.4	102.1 ± 1.7	101.3 ± 2.4	103.6 ± 1.7	103.5 ± 2.0	0.564			103.2 ± 8.2	103.2 ± 8.7	0.982
Length (mm)^‡^	165.6 ± 1.6	161.5 ± 1.8	166.0 ± 2.0	162.7 ± 1.8	163.3 ± 2.0	0.375			166.5 ± 7.6	161.1 ± 8.1	0.002
Width (mm)^‡^	134.6 ± 1.5	133.2 ± 1.5	129.6 ± 1.6	134.3 ± 1.5	133.9 ± 1.8	0.175			135.7 ± 7.1	130.5 ± 6.4	0.001
Width/Length^‡^	0.81 ± 0.01	0.83 ± 0.01	0.78 ± 0.01	0.83 ± 0.01	0.82 ± 0.01	0.018	c < b,d		0.82 ± 0.05	0.81 ± 0.04	0.649
Height/Length^‡^	0.64 ± 0.01	0.63 ± 0.01	0.61 ± 0.01	0.64 ± 0.01	0.63 ± 0.01	0.281			0.62 ± 0.04	0.64 ± 0.05	0.024
Height/Width^‡^	0.79 ± 0.01	0.77 ± 0.01	0.78 ± 0.01	0.77 ± 0.01	0.77 ± 0.01	0.794			0.76 ± 0.05	0.79 ± 0.05	0.004

MMSE, mini mental status examination

Each age group consisted of 10 men and 10 women. All values are presented as mean ± standard deviation

^*^ One-way analysis of variance with Bonferroni post-hoc comparisons

^†^ Paired *t*-test

**Fig. 1:**
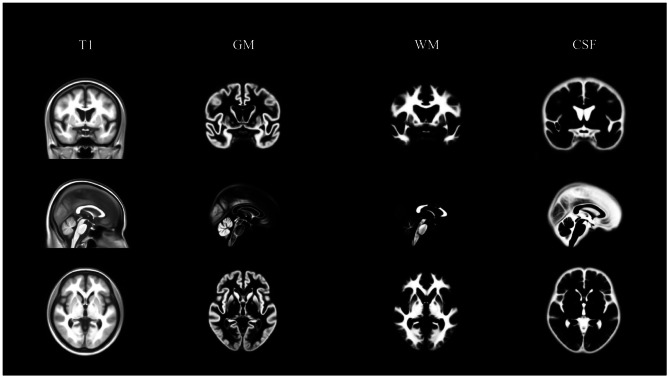
KNE200 brain MRI template

Figure 1 shows the KNE200 template, along with the probability map of each tissue class. Sex- and age-specific templates are shown in Supplementary Fig. 1.

### Effects of the sample size of templates on the deformations associated with spatial normalization

**Fig. 2:**
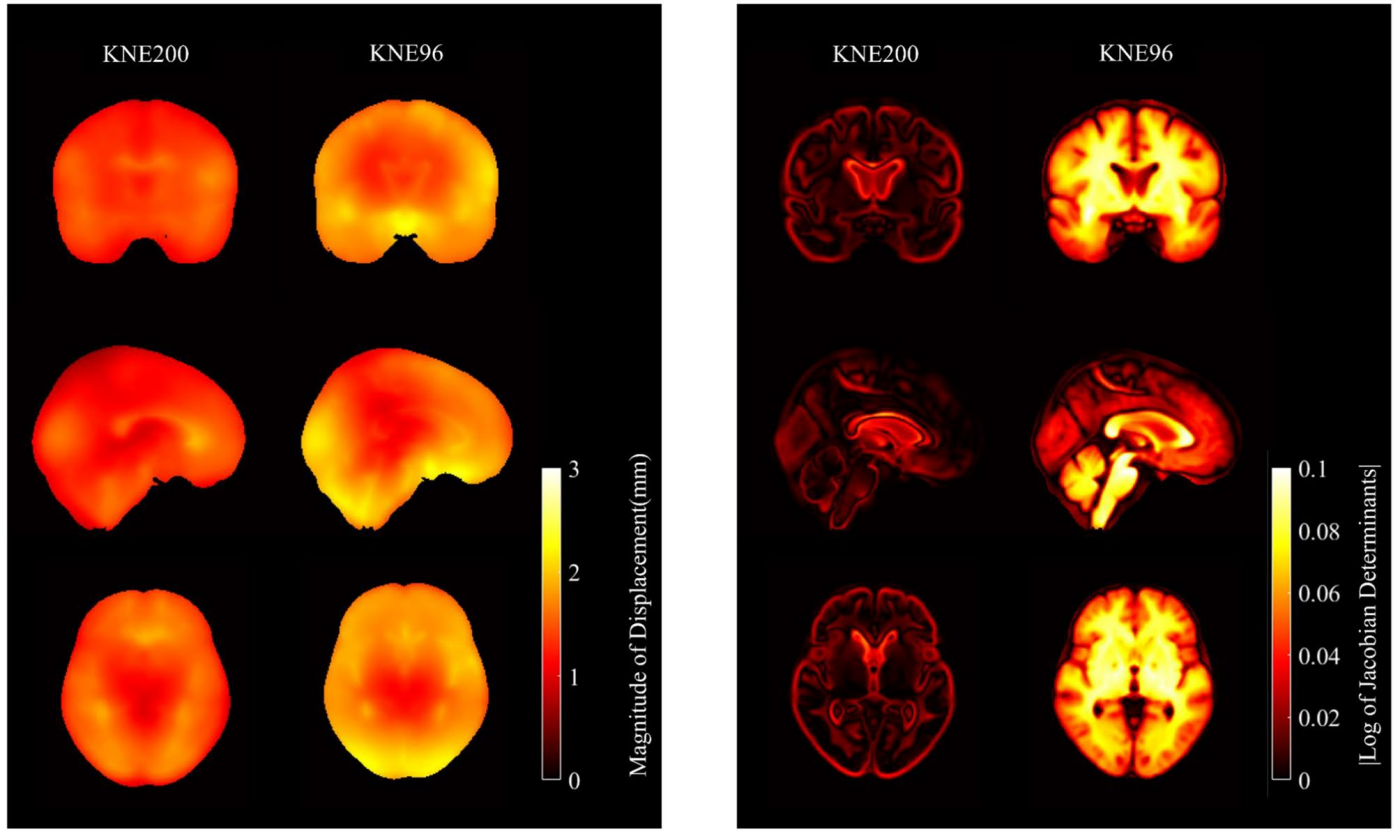
Average maps of validation dataset after registration to KNE200 and KNE96. **(A)** Maps of magnitude of distance. **(B)** Maps of |log of Jacobian determinants|

Figure 2 shows the maps of the deformation produced by spatial normalization to the templates in the MNI space. The map of the magnitude of displacement in Fig. 2A represents the Euclidean distance of each voxel between the original and registered images, and the map of the log values of the Jacobian determinants in Fig. 2B represents the volumetric change of each voxel after registration.

The KNE200 yielded significantly less displacement of voxels in 106 regions and less volumetric change in 122 regions compared to in the KNE96. There were no regions where the KNE200 yielded significantly more displacement or volumetric change of voxels compared to in the KNE96. The differences in the displacement and volumetric change of voxels due to spatial normalization between the KNE200 and the KNE96 were prominent in the parahippocampal gyrus, medial and posterior orbital gyrus, fusiform gyrus, gyrus rectus, and lobules of the cerebellum and vermis. The KNE200 induced at least 22% and up to 36% less displacement compared to the KNE96. Moreover, the KNE200 induced at least 18% and up to 46% less volumetric change compared to the KNE96 (Tables 3 and 4).

**Table 3:** Comparison of the displacement associated with spatial normalization between the KNE200 and the KNE96 templates

AAL3 labels	Displacement (mm)			Statistics^*^	
	KNE200	KNE96		*t*	*p*
Left parahippocampal gyrus	1.25 ± 0.48	1.74 ± 0.71		-11.45	7.72*10^− 21^
Lobule 4,5 of vermis	1.17 ± 0.42	1.52 ± 0.63		-10.46	1.10*10^− 17^
Left fusiform gyrus	1.29 ± 0.45	1.80 ± 0.80		-10.40	1.49*10^− 17^
Lobule 6 of vermis	1.28 ± 0.52	1.86 ± 0.95		-10.19	4.19*10^− 17^
Left lobule 6 of cerebellum	1.33 ± 0.48	1.87 ± 0.90		-9.67	5.63*10^− 16^
Left lobule 4,5 of cerebellum	1.24 ± 0.44	1.60 ± 0.65		-9.67	5.63*10^− 16^
Right lobule 6 of cerebellum	1.30 ± 0.52	1.87 ± 0.94		-9.47	1.60*10^− 15^
Left gyrus rectus	1.38 ± 0.55	2.02 ± 1.03		-9.46	1.67*10^− 15^
Left crus 1 of cerebellum	1.24 ± 0.54	1.89 ± 1.10		-9.40	2.26*10^− 15^
Left lobule 7b of cerebellum	1.12 ± 0.53	1.75 ± 1.06		-9.32	3.28*10^− 15^
Right lobule 4,5 of cerebellum	1.20 ± 0.45	1.58 ± 0.68		-9.22	5.51*10^− 15^
Left lobule 10 of cerebellum	1.29 ± 0.58	1.90 ± 1.00		-9.15	7.73*10^− 15^
Right crus 2 of cerebellum	1.09 ± 0.57	1.73 ± 1.14		-9.14	8.22*10^− 15^
Right fusiform gyrus	1.30 ± 0.47	1.79 ± 0.82		-9.13	8.64*10^− 15^
Left posterior orbital gyrus	1.37 ± 0.52	2.01 ± 1.00		-8.89	2.83*10^− 14^
Left crus 2 of cerebellum	1.16 ± 0.59	1.81 ± 1.21		-8.88	2.97*10^− 14^
Left medial orbital gyrus	1.35 ± 0.58	1.98 ± 1.10		-8.78	4.91*10^− 14^
Right parahippocampal gyrus	1.31 ± 0.43	1.74 ± 0.70		-8.61	1.20*10^− 13^
Right lobule 10 of cerebellum	1.24 ± 0.55	1.82 ± 1.00		-8.59	1.30*10^− 13^
Left lobule 9 of cerebellum	1.42 ± 0.57	2.01 ± 1.01		-8.56	1.51*10^− 13^
Right lobule 7b of cerebellum	1.02 ± 0.57	1.62 ± 1.13		-8.35	4.29*10^− 13^
Right lobule 8 of cerebellum	1.14 ± 0.54	1.73 ± 1.10		-8.05	1.89*10^− 12^
Right gyrus rectus	1.43 ± 0.52	2.01 ± 1.02		-7.99	2.53*10^− 12^
Right medial orbital gyrus	1.32 ± 0.50	1.82 ± 1.02		-6.69	1.37*10^− 9^
Right posterior orbital gyrus	1.36 ± 0.45	1.87 ± 0.94		-6.16	1.57*10^− 8^

Automated Anatomical Labelling Atlas 3

^*^ Paired *t*-test

**Table 4:** Comparison of the volumetric change associated with spatial normalization between the KNE200 and the KNE96 templates

AAL3 labels	|Log of Jacobian determinant|			Statistics^*^	
	KNE200	KNE96		*t*	*p*
Left posterior orbital gyrus	0.03 ± 0.01	0.04 ± 0.02		-12.09	3.38*10^− 21^
Left medial orbital gyrus	0.03 ± 0.01	0.04 ± 0.02		-9.89	1.86*10^− 16^
Right lobule 8 of cerebellum	0.03 ± 0.01	0.04 ± 0.02		-9.42	2.04*10^− 15^
Lobule 4,5 of vermis	0.04 ± 0.02	0.07 ± 0.05		-9.17	7.02*10^− 15^
Left lobule 7b of cerebellum	0.04 ± 0.02	0.06 ± 0.04		-9.10	1.01*10^− 14^
Right lobule 10 of cerebellum	0.04 ± 0.02	0.06 ± 0.04		-9.05	1.31*10^− 14^
Lobule 6 of vermis	0.05 ± 0.02	0.07 ± 0.04		-8.80	4.47*10^− 14^
Right posterior orbital gyrus	0.02 ± 0.01	0.03 ± 0.02		-8.78	4.94*10^− 14^
Left crus 1 of cerebellum	0.03 ± 0.01	0.05 ± 0.03		-8.68	8.26*10^− 14^
Left lobule 10 of cerebellum	0.04 ± 0.02	0.07 ± 0.04		-8.44	2.66*10^− 14^
Left crus 2 of cerebellum	0.04 ± 0.02	0.07 ± 0.04		-8.35	4.23*10^− 13^
Right lobule 7b of cerebellum	0.03 ± 0.02	0.06 ± 0.04		-8.34	4.46*10^− 13^
Left lobule 6 of cerebellum	0.03 ± 0.01	0.04 ± 0.03		-8.23	7.77*10^− 13^
Right Fusiform gyrus	0.04 ± 0.02	0.06 ± 0.03		-8.20	9.07*10^− 13^
Left gyrus rectus	0.04 ± 0.02	0.05 ± 0.03		-8.11	1.43*10^− 13^
Right lobule 9 of cerebellum	0.05 ± 0.03	0.07 ± 0.04		-8.01	2.31*10^− 12^
Right crus 2 of cerebellum	0.04 ± 0.02	0.07 ± 0.04		-7.87	4.50*10^− 12^
Left lobule 4,5 of cerebellum	0.02 ± 0.01	0.03 ± 0.02		-7.83	5.47*10^− 12^
Right gyrus rectus	0.04 ± 0.02	0.05 ± 0.03		-7.62	1.53*10^− 11^
Left parahippocampal gyrus	0.04 ± 0.02	0.05 ± 0.03		-7.53	2.46*10^− 11^
Right parahippocampal gyrus	0.04 ± 0.02	0.06 ± 0.04		-7.50	2.84*10^− 11^
Right lobule 6 of cerebellum	0.03 ± 0.01	0.04 ± 0.03		-7.42	4.13*10^− 11^
Left fusiform gyrus	0.02 ± 0.01	0.03 ± 0.02		-6.90	5.04*10^− 10^
Right medial orbital gyrus	0.02 ± 0.01	0.03 ± 0.01		-6.87	5.76*10^− 10^
Right lobule 4,5 of cerebellum	0.02 ± 0.01	0.03 ± 0.02		-6.47	3.69*10^− 9^

Automated Anatomical Labelling Atlas 3

^*^ Paired *t*-test

### Effects of the sample ethnicity of the templates on the deformation associated with normalization

**Fig. 3:**
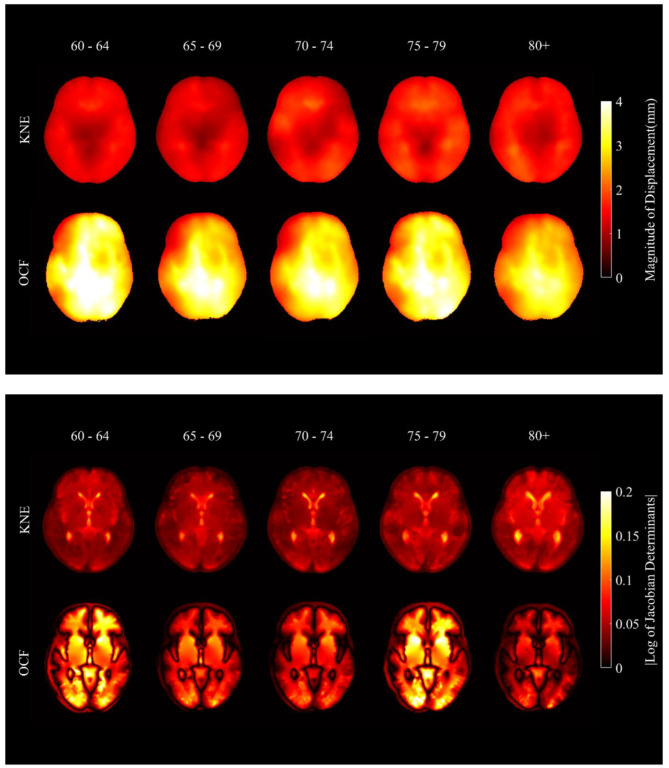
Average maps of validation dataset after registration to age-specific templates of KNE200 and OCF. **(A)** Maps of magnitude of distance. **(B)** Maps of |log of Jacobian determinants|

Figure 3 shows the maps of the deformation produced by spatial normalization to the age-specific templates of the KNE200 and the OCF in the MNI space. The KNE200 yielded significantly less displacement of voxels in all age groups (115 regions in the 60–64-year-old group, 114 regions in the 65–69-year-old group, 103 regions in the 70–74-year-old group, 111 regions in the 74–79-year-old group, and 80 regions in the 80 years or older group) compared to the OCF. It also exhibited less volumetric changes (144 regions in the 60–64-year-old group, 21 regions in the 65–69-year-old group, 15 regions in the 70–74-year-old group, 62 regions in the 74–79-year-old group, and 9 regions in the 80 years old or above group) compared to the OCF. The KNE200 did not yield significantly more displacement of voxels compared to the OCF in any regions across all age groups. In addition, the KNE200 did not yield significantly more volumetric changes of voxels compared to the OCF in any regions in age groups under 80 years. However, in the 80 years old or above group, the KNE200 yielded more volumetric changes than the OCF in two regions: the right paracentral lobule (t = 5.153, *p* = 5.66*10 − 5) and the left postcentral gyrus (t = 4.846, *p* = 1.12*10 − 4).

In all age groups, the differences in the displacement of voxels due to spatial normalization between the KNE200 and the OCF were prominent in the cerebellum, vermis, thalamus, hippocampus, and parahippocampal gyrus. The KNE200 induced at least 58% and up to 78% less displacement compared to the OCF (Table 5).

**Table 5:** Comparison of the displacement associated with spatial normalization between the KNE200 and the OCF templates

AAL3 labels	Displacement (mm)			Statistics^*^	
	KNE200	OCF		*t*	*p*
60–64 years old					
Right lobule 4,5 of cerebellum	1.13 ± 0.48	4.82 ± 0.45		-26.26	2.15*10^− 16^
Lobule 4,5 of vermis	1.05 ± 0.41	4.21 ± 0.41		-25.27	4.36*10^− 16^
Right medial pulvinar nucleus thalamus	0.90 ± 0.35	3.83 ± 0.59		-23.26	2.01*10^− 15^
Right parahippocampal gyrus	1.22 ± 0.34	3.57 ± 0.40		-18.99	8.12*10^− 14^
Right hippocampus	1.26 ± 0.41	4.03 ± 0.50		-16.83	7.17*10^− 13^
65–69 years old					
Right ventral posterolateral nucleus thalamus	0.97 ± 0.27	3.73 ± 0.51		-25.90	2.76*10^− 17^
Lobule 4,5 of vermis	1.10 ± 0.43	3.86 ± 0.46		-21.90	6.11*10^− 15^
Right lobule 4,5 of cerebellum	1.10 ± 0.46	3.98 ± 0.46		-19.69	4.22*10^− 14^
Right hippocampus	1.33 ± 0.29	3.57 ± 0.53		-17.76	2.74*10^− 13^
Right parahippocampal gyrus	1.29 ± 0.39	3.19 ± 0.42		-15.04	5.24*10^− 12^
70–74 years old					
Right lobule 4,5 of cerebellum	1.18 ± 0.36	3.93 ± 0.41		-25.86	2.86*10^− 16^
Right hippocampus	1.43 ± 0.43	3.55 ± 0.41		-23.86	1.26*10^− 15^
Lobule 4,5 of vermis	1.17 ± 0.35	3.84 ± 0.36		-22.48	3.77*10^− 15^
Right parahippocampal gyrus	1.37 ± 0.43	3.19 ± 0.40		-20.74	1.64*10^− 14^
Right medial pulvinar nucleus thalamus	1.23 ± 0.36	3.56 ± 0.37		-19.61	4.56*10^− 14^
75–79 years old					
Right medial pulvinar nucleus thalamus	1.17 ± 0.38	3.50 ± 0.47		-24.25	9.36*10^− 16^
Lobule 4,5 of vermis	1.20 ± 0.42	3.84 ± 0.38		-18.70	1.08*10^− 13^
Right lobule 4,5 of cerebellum	1.32 ± 0.46	4.30 ± 0.51		-17.41	3.88*10^− 16^
Right parahippocampal gyrus	1.38 ± 0.55	3.31 ± 0.49		-11.64	4.34*10^− 10^
Right hippocampus	1.50 ± 0.59	3.63 ± 0.67		-10.97	1.16*10^− 9^
80 + years old					
Right lobule 4,5 of cerebellum	1.22 ± 0.43	3.75 ± 0.59		-15.18	4.46*10^− 12^
Right parahippocampal gyrus	1.24 ± 0.40	3.06 ± 0.48		-13.10	5.79*10^− 11^
Lobule 4,5 of vermis	1.35 ± 0.46	3.44 ± 0.64		-12.94	7.18*10^− 11^
Right hippocampus	1.40 ± 0.51	3.32 ± 0.57		-12.46	1.36*10^− 10^
Right ventral posterolateral nucleus thalamus	1.34 ± 0.35	3.32 ± 0.58		-12.25	1.84*10^− 10^

Automated Anatomical Labelling Atlas 3

^*^ Paired *t*-test

The differences in the volumetric change of voxels due to spatial normalization between the KNE200 and the OCF were also prominent in the cerebellum, vermis, hippocampus, and parahippocampal gyrus in the age groups under 80 years old and in the cerebellum and vermis regions of the 80 years or older group. The KNE200 induced at least 37% and up to 77% less volumetric change compared to the OCF (Table 6).

**Table 6:** Comparison of the volumetric change associated with spatial normalization between the KNE200 and the OCF templates

AAL3 labels	|Log of Jacobian determinant|			Statistics^*^	
	KNE200	OCF		*t*	*p*
60–64 years old					
Right lobule 4,5 of cerebellum	0.04 ± 0.02	0.16 ± 0.03		-19.68	4.28*10^− 14^
Lobule 4,5 of vermis	0.04 ± 0.02	0.14 ± 0.02		-18.97	8.28*10^− 14^
Left hippocampus	0.05 ± 0.02	0.17 ± 0.03		-15.98	1.79*10^− 12^
Left parahippocampal gyrus	0.04 ± 0.01	0.10 ± 0.02		-13.77	2.45*10^− 11^
Left ventral anterior nucleus thalamus	0.06 ± 0.03	0.13 ± 0.06		-5.00	7.91*10^− 5^
65–69 years old					
Right amygdala	0.03 ± 0.02	0.09 ± 0.02		-9.00	2.78*10^− 8^
Left hippocampus	0.04 ± 0.02	0.10 ± 0.02		-8.57	6.00*10^− 8^
Right lobule 6 of cerebellum	0.03 ± 0.02	0.10 ± 0.02		-7.87	2.15*10^− 7^
Left parahippocampal gyrus	0.03 ± 0.02	0.06 ± 0.02		-6.44	3.61*10^− 6^
Lobule 4,5 of vermis	0.05 ± 0.03	0.09 ± 0.02		-4.64	1.77*10^− 4^
70–74 years old					
Left lobule 10 of cerebellum	0.02 ± 0.01	0.06 ± 0.01		-8.87	3.47*10^− 8^
Right lobule 4,5 of cerebellum	0.03 ± 0.02	0.09 ± 0.03		-6.87	1.49*10^− 6^
Left parahippocampal gyrus	0.03 ± 0.01	0.07 ± 0.02		-6.39	3.97*10^− 6^
Left hippocampus	0.04 ± 0.02	0.09 ± 0.03		-6.07	7.81*10^− 6^
Lobule 6 of vermis	0.03 ± 0.02	0.07 ± 0.02		-4.73	1.47*10^− 4^
75–79 years old					
Left lobule 8 of cerebellum	0.02 ± 0.01	0.06 ± 0.01		-11.98	2.66*10^− 10^
Right lobule 4,5 of cerebellum	0.04 ± 0.02	0.15 ± 0.04		-10.94	1.21*10^− 9^
Lobule 4,5 of vermis	0.04 ± 0.02	0.13 ± 0.02		-9.68	8.94*10^− 9^
Left hippocampus	0.05 ± 0.02	0.17 ± 0.05		-9.30	1.68*10^− 8^
Left parahippocampal gyrus	0.04 ± 0.02	0.12 ± 0.03		-8.87	3.48*10^− 8^
80 + years old					
Left lobule 10 of cerebellum	0.03 ± 0.01	0.06 ± 0.02		-7.08	9.73*10^− 7^
Left lobule 4,5 of cerebellum	0.04 ± 0.02	0.09 ± 0.03		-6.40	3.85*10^− 6^
Lobule 4,5 of vermis	0.05 ± 0.03	0.08 ± 0.02		-5.81	1.34*10^− 5^
Right lobule 6 of cerebellum	0.04 ± 0.02	0.10 ± 0.04		-5.80	1.37*10^− 5^
Left lobule 6 of cerebellum	0.04 ± 0.02	0.10 ± 0.04		-5.79	1.40*10^− 5^

Automated Anatomical Labelling Atlas 3

^*^ Paired *t*-test

## Discussion

This study developed and validated a standardized brain MRI template of cognitively normal elderly Koreans, the KNE200, using age- and sex-balanced datasets that included high-resolution 3.0T T1 structural brain magnetic resonance images. Brain shape and size vary among age, sex, and race, and to improve the accuracy of spatial normalization to a standard template in neuroimaging studies, many population-specific brain templates have been created [30, 31]. However, in many studies, templates were developed from datasets where the age and sex of subjects were not strictly balanced [10, 13–15]. The amount of gray and white matter decreases with age, and women tend to have smaller but higher ratios of gray matter than men [32, 33]. Moreover, in young adults, the brains of different races and ethnic groups differ [9, 10]. Therefore, it is important to use population-specific templates. If errors due to spatial normalization decrease, the accuracy of other consequent image processing procedures may also improve. Previous studies have shown that the segmentation of tissues improved when study-specific templates were used rather than the MNI template [5, 6].

The quality of the brain MRI template was considerably influenced by the sample size. The deformation of voxels associated with spatial normalization asymptotically decreased as the sample size increased until the sample size reached 200 [7]. For this reason, we developed the KNE200, although we had already developed the KNE96, and as expected, both the displacement and volumetric change of voxels occurred significantly less when images were normalized to the KNE200 as opposed to the KNE96 [18]. In particular, in areas with large individual differences in shape and size, such as the parahippocampal gyrus or cerebellum, it is important to minimize the deformation associated with spatial normalization using a template with a sufficient sample size. The parahippocampal gyrus is a region with great variability in shape and size because of the collateral sulcus; in addition, the cerebellum also varies greatly in shape and asymmetry between individuals [34, 35]. This study directly demonstrated that we could significantly reduce the displacement and volumetric change of voxels in these regions by employing the KNE200 instead of the KNE96 as a template for spatial normalization. By taking the inverse log of the Jacobian determinants, we can calculate the amount of volumetric change associated with spatial normalization in these regions. The volume increase of the parahippocampal gyrus after spatial normalization to the KNE200 was only about one-seventh of that of the KNE96 (33 mm3 for the KNE200 and 247 mm3 for the KNE96). Considering that the mean volume of the parahippocampal gyrus was 5700 mm3 in normal controls and 5500 mm3 in patients with mild cognitive impairment (MCI), the difference in the volume increases of the parahippocampal gyrus associated with spatial normalization between the KNE200 and the KNE96, which was higher than 200 mm3, is enough to confound the differences in the volume of the parahippocampal gyrus between normal controls and patients with MCI [36]. The volume increase of the cerebellum after spatial normalization to the KNE200 was only about one-eighth of that of the KNE96 (494 mm3 for the KNE200 and 4,272 mm3 for the KNE96). Considering that the difference in the mean volume of the cerebellum between normal controls and MCI patients is minimal (123,249 mm3 in normal controls and 122,394 mm3 in MCI patients), the difference in the volume increase of the cerebellum associated with spatial normalization between the KNE200 and the KNE96 is large [37].

Several studies have reported that ethnic-specific templates can reduce the deformation associated with spatial normalization [7, 13, 18]. In Chinese subjects, the Chinese2020 template, made from Chinese subjects aged 18–76 years, showed less deformation than the MNI152 [13]. In Koreans, the KNE96 template, made from normal elderly Koreans aged over 60 years, showed significantly less deformation after spatial normalization in the frontal gyrus, occipital gyrus, caudate, and thalamus compared to in the MNI152 [18]. However, the age distributions of the samples employed in the construction of the templates were not comparable. Since brain morphology changes with advancing age, the differences in the deformation between the templates may not be solely attributable to the different ethnicities of the templates [12]. In young adults, the accuracy of image registration and tissue segmentation increases when an ethnicity-matched template is employed [7]. The templates constructed from young Chinese subjects showed the greatest anatomical differences in the right supramarginal gyrus, inferior frontal gyrus, and superior temporal gyrus compared to those constructed from young Caucasians, indicating that ethnicity-matched templates may reduce deformations associated with spatial normalization [7]. The current study clearly demonstrated that ethnic-specific templates may reduce the deformation associated with spatial normalization in older adults. In particular, when the KNE200 was employed in the spatial normalization instead of the OCF, deformation occurred significantly less after spatial normalization in the parahippocampal gyrus, where East Asians typically have larger volumes compared to Caucasians [8, 9]. However, older Caucasian adults showed larger volumes in the parahippocampal gyrus than older East Asian adults [38]. In addition, deformation of the hippocampus also occurred less when the KNE200 was employed in the spatial normalization as opposed to the OCF. Although the volume of the hippocampus was comparable in young adults between East Asians and Caucasians, age-associated volume loss of the hippocampus was slower in East Asians than in Caucasians [9, 39]. Because ethnicity affects brain morphology and contributes differentially to age-associated brain atrophy in normal older adults, it is important to use ethnicity-matched brain templates in neuroimaging studies of elderly populations [9, 40].

The statistical power of neuroimaging studies depends on image registration errors [41]. Therefore, to increase the statistical power of neuroimaging studies, accurate image registration is crucial. Accurate spatial normalization leads to increased sensitivity and the prevention of false negatives in neuroimaging studies [42]. For example, voxel-based morphometry (VBM) is a method that extracts feature such as signal intensity from all three tissue types of T1 MRI scans to detect brain atrophy [43–45]. Because the detection accuracy of VBM depends on the results of spatial normalization, choosing the right template for VBM methodology may increase the chance of accurate detection of structural differences between groups [46, 47]. By using the KNE200 template, spatial normalization accuracy will improve, and the results of VBM studies of elderly Koreans on hippocampal atrophy may be more accurate. Moreover, T1 MRI brain templates are used to compare and combine findings from different imaging modalities, such as positron emission tomography (PET) [48]. When native T1 images were spatially normalized to the T1 template, the deformation fields were calculated and reapplied to PET scans, which were co-registered with native T1 images [48]. In fluorine-18-fluorodeoxy-D-glucose-PET (FDG-PET) studies, patients with Alzheimer’s disease show hypometabolism in the hippocampus [49, 50]. However, because PET scans lack structural information, structural MRI, such as T1, is used in the quantitative analysis of PET [51]. Therefore, accurate spatial normalization and segmentation of brain areas are important in PET studies. Choosing the appropriate T1 template can lead to more accurate results in the anatomical localization of PET scans, thereby increasing the power of the statistical analysis [48, 52]. Statistical power is usually expressed using sample sizes, representing the number of subjects that should be recruited for a given study [41]. Therefore, by using the right T1 template in neuroimaging studies, studies can benefit from smaller samples, resulting in smaller study costs and shorter study times.

Although the KNE200 produced far less deformation of voxels after spatial normalization than the OCF in older Koreans, the difference was reduced in older adults aged 80 years or older. The number of regions that showed less displacement after spatial normalization to the KNE200 compared to the OCF was over 100 regions in Koreans aged 60–79 years and 80 regions in those aged 80 years or older. The number of regions that showed less volumetric change after spatial normalization to the KNE200 than to the OCF was 15–144 in Koreans aged 60–79 years and 9 in those aged 80 years or older. This difference may be partly attributable to the acceleration of age-associated brain atrophy in older adults aged 80 years or older. According to Allen and colleagues, the rates of volume loss in gray matter and white matter were 9.1–9.8% and 5.6–6.4% respectively between the ages of 30 and 70 years and 11.3–12.3% and 21.6–25.0% between the ages of 30 and 80 years [53]. The faster volume loss of the brain may mitigate the ethnic differences in brain volume in older adults aged 80 years or older. In older adults aged 80 years or older, the KNE200 produced more deformation after spatial normalization in the paracentral lobule and postcentral gyrus than the OCF. These regions are known to be relatively resilient to aging and to vary less among individuals [54, 55]. Because both age-specific KNE200 and OCF templates were constructed from small samples, both templates may contain variability in these regions.

This study has several limitations. First, we compared the KNE200 with the OCF to examine the effect of the ethnicity of the brain template on the deformation associated with spatial normalization. However, the participants employed in the construction of the OCF included older adults without dementia, while those in the construction of the KNE200 included cognitively normal older adults only. Therefore, the differences in the deformation associated with spatial normalization between the two templates might have been influenced by the participants with prodromal dementing illnesses that were potentially included in the development of the OCF. To the best of our knowledge, brain MRI templates constructed from cognitively normal older Caucasians have not yet been reported. Second, the number of individuals included in the development of age-specific templates was limited. The age-specific templates of KNE200 were constructed from only 40 individuals. In addition, the number of individuals in each age group of the OCF dataset was not balanced and varied from 36 to 101. Additionally, our study is limited by the uniform nature of our dataset, especially in terms of cognitive abilities, gender distribution, and educational background. This underscores the need for further validation across diverse groups and for assessing its effectiveness in identifying disease-specific changes. Enhancing the robustness and universal applicability of the KNE200 template is essential, as it will enable broader application in a variety of clinical and research settings.

In conclusion, KNE200 can reduce the deformation associated with spatial normalization in older Koreans.

### Electronic supplementary material

Below is the link to the electronic supplementary material.

Supplementary Material 1

## Data Availability

The datasets used and/or analyzed during the current study are available from the corresponding author on reasonable request.
