# Supplementary figures and images for: Construction and validation of a brain magnetic resonance imaging template for normal older Koreans
Source: BMC Neurol. 2024 Jun 28;24:222. doi: 10.1186/s12883-024-03735-8 (PMC11212263; doi:10.1186/s12883-024-03735-8)

**Supplementary Figure 1**. Age- and sex-specific templates of KNE200.

**
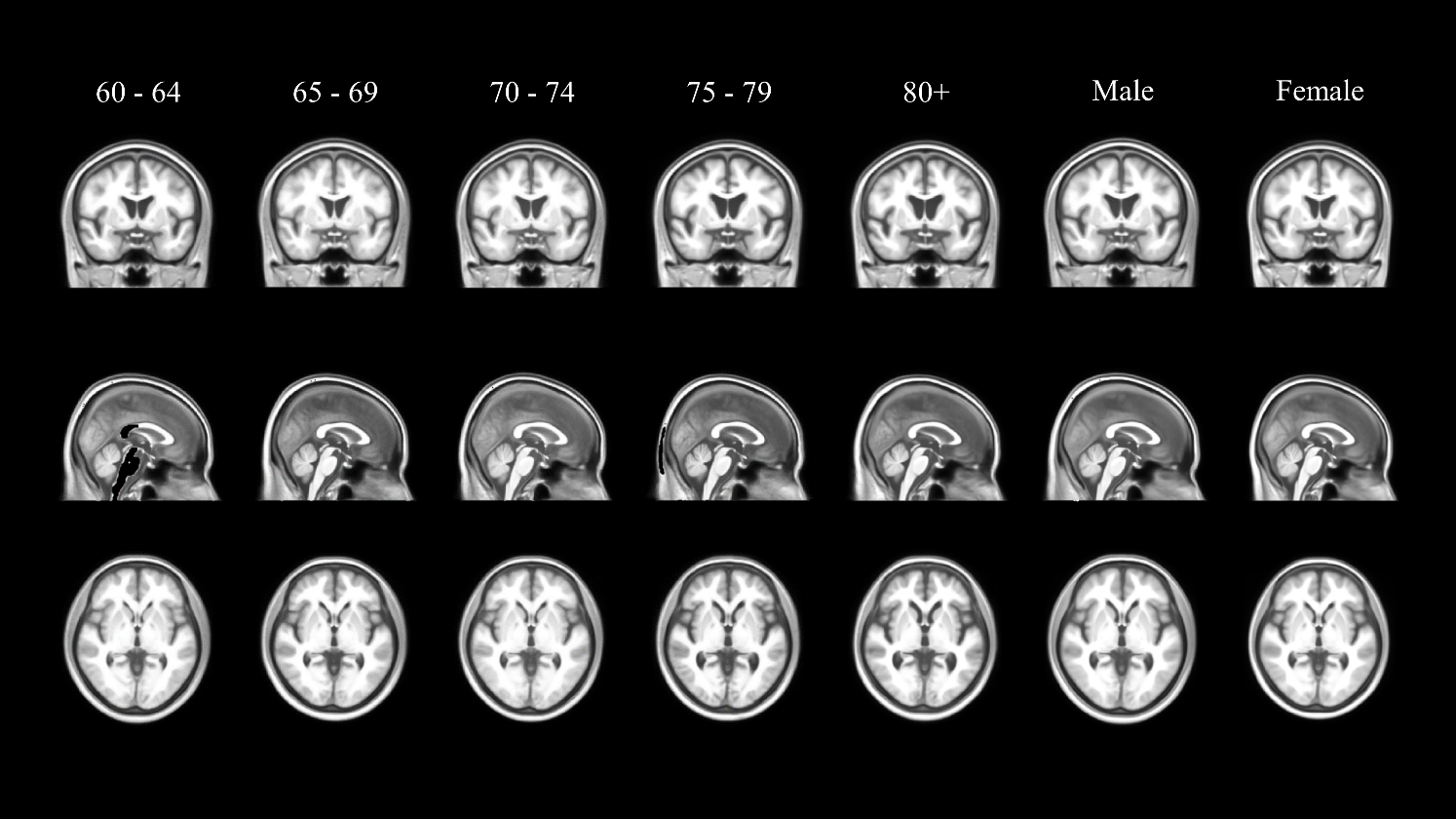
**

Supplement: Supplementary file 1 — Supplementary Material 1 [file 12883_2024_3735_MOESM1_ESM.docx]
